# The Treatment of Pneumonia

**Published:** 1869-09

**Authors:** 


### The Treatment of Pneumonia.

Dr. J. Hughes Bennett has an article in the *Practitioner* reviewing the restorative treatment of pneumonia, and contrasting the results with those obtained by other methods of treatment, particularly that recommended by Dr. Richardson. Regarding the management of acute pneumonia Dr. Bennett considers the following axioms fully established:

1. The great end of medical practice is to remove the consolidation of the lungs, and restore those organs to their natural condition as rapidly as possible.

2. To this end everything that diminishes vital strength should be avoided, and nutrients administered as early as possible, to favor the cell transformation necessary for removing the exudation from the lungs.

3. There is no relation between the violence of the symptoms or force of the pulse, and the fatality of the disease. Young and vigorous subjects suffer most, but almost recover soonest.

4. The weak pulse, want of reaction, non-disappearance of the pneumonic consolidation, or its appearance during the progress of exhaustive diseases, are the unfavorable signs of pneumonia.

5. Continued exercise or work after the attack; low diet; large blood-lettings; depressants, such as tartar-emetic and sedatives; expectorants, such as squills and ipecacuanha; mercury and violent purgatives, are opposed to the restorative treatment of the disease, and when not fatal, tend to prolong its duration.

6. Small blood-lettings of from six to eight ounces may be used in extreme cases, more especially of double pneumonia or of broncho-pneumonia, as a palliative to relieve tension of the blood-vessels and congestion of the right heart and lungs.

7. Local pain is best relieved by large warm poultices.

8. The true disease, that is, the exudation which has infiltrated itself through the pulmonary tissues and been coagulated, constituting hepatization, can only be removed, first, by its transformation into pus cells; second, by the molecular degeneration and liquefaction of these; third, by absorption into the blood; and fourth, by excretion of the exuded matter in a chemically altered form through the evacuations.

9. These processes are favored by supporting the vital powers:

first, by rest in bed immediately after the attack; second, by beef-tea and milk during the febrile period, with a moderate amount of wine, if the pulse be feeble; third, by beef-steaks and solid food as soon as they can be taken, with more wine or a little spirits, if the pulse falter; fourth, by mild diuretics on the seventh or eighth day, to favor excretion by the kidneys.

10. The same pathology and principles of treatment apply to all cases of simple pneumonia, whether single or double, the latter being only modified by the weakness of the patient, when more restoratives and stimulants are required,

11. In complicated cases other treatment may be required according to the circumstances of the case; the pneumonia, however, being always influenced in the manner previously detailed.

The mortality in 153 cases treated on this plan was one death in 30½ cases. This statement includes 35 double and 24 complicated cases. Among the simple cases, single or double, the mortality was *nil.—Medical Record.*

